# sTNFR-II and sICAM-1 are associated with acute disease and hepatic inflammation in schistosomiasis japonica

**DOI:** 10.1016/j.ijpara.2007.09.013

**Published:** 2008-05

**Authors:** Magda K. Ellis, Yuesheng Li, Xunya Hou, Honggen Chen, Donald P. McManus

**Affiliations:** a Molecular Parasitology Laboratory, Australian Centre for International and Tropical Health and Nutrition, The Queensland Institute of Medical Research, The University of Queensland, 300 Herston Road, Herston, Brisbane, Qld 4029, Australia; b Hunan Institute of Parasitic Diseases, WHO Collaborating Centre for Research and Control on Schistosomiasis in Lake Region, Yueyang, Hunan Province, PR China; c Jiangxi Provincial Institute of Parasitic Diseases, 330046 Nanchang, PR China

**Keywords:** Schistosomiasis japonica, Hepatic inflammation, TNFRs, sICAM-1, IgG antibodies

## Abstract

Soluble intracellular adhesive molecule 1 (sICAM-1) and tumour necrosis factor receptors I (TNFR-1) and II (TNFR-II) have been shown to be associated with numerous liver disorders. Shedding of these membrane proteins can be triggered by the Th1 cytokines, TNF-alpha and IFN-gamma, which are associated with susceptibility or resistance to hepatic schistosomiasis, respectively. Further, TNF-alpha receptors and sICAM-1 have been implicated in periportal fibrosis in advanced human schistosomiasis mansoni and correlate with schistosome granuloma formation in the murine model. We measured serum levels of sICAM-1, TNFR-I and TNFR-II in Chinese patients with different clinically defined stages of schistosomiasis japonica and controls; these included 35 patients with acute schistosomiasis, 45 patients with chronic schistosome infections, 34 advanced patients with evidence of severe morbidity and 20 patients with no known history of exposure to infection. Markedly elevated levels of soluble TNFRs (sTNFRs) and sICAM-1 were observed in the acute and advanced patients compared with the chronic and control groups. Mean sTNFR-II levels were significantly higher in acute patients compared with advanced (*P* < 0.00001) and chronic patients (*P* < 0.00001) and showed the strongest association of the markers with acute disease (odds ratio (OR) = 1.099). sTNFR-II and sICAM-1 levels both correlated with infection intensity and there were significant positive correlations observed between eosinophil count and infection intensity (*P* = 0.0072) and sICAM-1 (*P* = 0.0014). Although there were significantly higher levels of antigen-specific IgG4 and total IgG in infected individuals compared with controls, none correlated with infection intensity. Further, no differences in IgG4 and total IgG levels were observed between the acute and chronic groups. The results suggest sTNFRs and sICAM-1 are associated with liver inflammation and disease progression. Measurement of sTNFR-II and sICAM-1 levels in serum could serve as additional markers for the diagnosis of acute stage disease and the monitoring of hepatic inflammation in human schistosomiasis japonica.

## 1 Introduction

Schistosomiasis japonica, caused by *Schistosoma japonicum*, is known to have several clinical stages of disease which are a direct result of an immunopathological reaction to the deposition of eggs in vital organs of the host, particularly the liver and spleen. The eggs initiate the onset of acute infection characterised by non-specific symptoms including fever and eosinophilia and in some cases hepato-splenic inflammation and mild forms of diffuse fibrosis (Ross et al., 2007). Long-term effects of infection or repeated re-infection and subsequent accumulation of the egg-induced granuloma in the liver can be observed in chronic and, more predominantly, advanced patients. The latter typically display signs of severe liver damage, a result of large collagen deposits in the periportal spaces leading to portal hypertension, splenomegaly, shunting and gastrointestinal varices (Ross et al., 2002; Gryseels et al., 2006).

Granuloma formation is controlled and modulated by several cell types and protein interactions, primarily CD4+ T-cells, Th1 cytokines and cell adhesion factors (Wynn and Cheever, 1995; Jacobs and Van Marck, 1998; Stadecker et al., 2004). Intracellular adhesion molecule 1 (ICAM-1) is present on endothelial cells, antigen presenting cells and fibroblasts and belongs to the immunoglobulin superfamily of proteins (Springer, 1990; Jacobs and Van Marck, 1998; Zaremba and Losy, 2002). It is well established that the Th1 cytokines, IFN-gamma and TNF-alpha, both trigger the release of soluble ICAM-1 (sICAM-1) (Momosaki et al., 1995; Paolieri et al., 1997; Leung, 1999) and are known to play key roles in the modulation (Dessein et al., 2004; Bonnard et al., 2006) and aggravation of hepatic fibrosis (Henri et al., 2002; Booth et al., 2004), respectively. The soluble form of ICAM-1 is involved in lymphocyte and eosinophil recruitment and inflammatory, immune-mediated mechanisms (Kyan-Aung et al., 1991; Radi et al., 2001; Forbes et al., 2006). Raised levels of sICAM-1 have been observed in the serum of patients with acute and chronic liver disorders (Abdalla et al., 2002). Elevated expression has also been associated with intestinal schistosome infection and early granuloma formation in murine studies (Ritter et al., 1993; Ritter and McKerrow, 1996; El-Ahwany et al., 2000; Hassanein et al., 2001). sICAM-1 also plays a role in modulation of the schistosome granuloma (Jacobs and Van Marck, 1998). Further, the granulomatous response is mediated by eosinophils, the recruitment of which is critically dependent on ICAM-1 (Teixeira et al., 2001; Forbes et al., 2006). Human studies have also noted a significant increase in sICAM-1 levels in serum and plasma of hepatic schistosomiasis patients (Secor et al., 1994; Esterre et al., 1998; Mwatha et al., 1998).

TNF-alpha is thought to aggravate hepatic fibrosis caused by *Schistosoma mansoni* (Henri et al., 2002; Booth et al., 2004). TNF-alpha initiates shedding of the membrane receptors TNFR-I and II which are cleaved from the membrane and are detectable in serum, plasma and urine (Fernandez-Botran, 1999; Fernandez-Real et al., 1999; Dri et al., 2000). Given this relationship, levels of soluble TNFRs (sTNFRs) could reflect TNF-alpha activity. As receptor levels remain elevated longer than TNF-alpha, they have shown potential as markers of disease progression in human schistosomiasis (Mwatha et al., 1998) and have been implicated in schistosome oviposition and circumoval granuloma formation in murine studies (Haseeb et al., 2001).

Immunoglobulins IgG and subclass IgG4 have been shown to have a pivotal role in the humoral response to schistosomal infection. A few studies have reported that measurement of these antibodies can be used to differentiate between different disease stages, in particular acute and chronic patients (Kirinoki et al., 2003; Beck et al., 2004). High levels of IgG4 have also been associated with periportal fibrosis and portal hypertension in patients with advanced schistosomiasis mansoni (Tawfeek et al., 2003).

Here, we report on the serum levels of sICAM-1, sTNFR-I and sTNFR-II in patients with different clinically defined stages of schistosomiasis japonica as the basis for investigating their role in schistosome-induced human hepatic disease. In addition, total IgG and IgG4 levels were assessed to investigate their potential in the differential diagnosis of disease stage.

## 2 Materials and methods

### 2.1 Study patients

The study involved 127 participants from endemic areas of the Poyang and Dongting lakes in Jiangxi and Hunan provinces, China, respectively, with different clinical stages of schistosomiasis (Table 1), defined according to the guidelines established by the Ministry of Public Health in China (Chen and Mott, 1988; MPHC, 2001).

Thirty-five subjects were diagnosed with acute disease. These individuals had all tested egg-positive after stool examination using the Kato-Katz (Katz et al., 1972) thick smear technique, by serology using indirect haemagluttination and ELISA assays with soluble egg antigen (SEA) and soluble worm adult protein (SWAP) (MPHC, 2001). They also had a clear history of recent water contact and presented clinical symptoms associated with acute infection including fever, cough, bloody diarrhoea and eosinophilia (⩾15% of their total leukocyte count). Forty-five patients with chronic schistosome infections also participated in the study. They were defined as individuals who were found to be infected with *S. japonicum* by faecal examination (Katz et al., 1972) during a population survey. They had a history of water contact and *S. japonicum* infection but were asymptomatic with no clinical features of disease. Advanced patients (*N* = 34) were diagnosed if they presented with splenomegaly (Hackett degree ⩾ II) (WHO, 2000. Ultrasound in schistosomiasis: A practical guide to the standardised use of ultrasonography for the assessment of schistosomiasis-related morbidity. In: Richter, J., Hatz, C., Campagne, G., Bergquist, N.R., Jenkins, J.M. (Ed). Second International Workshop, Niamey, Niger), hypersplenism and portal hypertension.

In addition, 20 subjects from non-endemic areas with no known history of exposure to infection or liver disease were recruited during routine health checks at a local clinic in Yueyang, Hunan province. All control subjects tested negative for *S. japonicum* infection using the Kato-Katz thick smear technique (Katz et al., 1972).

### 2.2 Serum processing

Venous blood samples (4–5 ml) were obtained from all subjects with informed consent. Sera were separated within 12 h of collection, using standard procedures and stored at −80 °C. Aliquots of all serum samples were then transported on dry ice to the Queensland Institute of Medical Research, Brisbane and stored at −80 °C until analysed. An additional 0.5 ml of blood was collected into EDTA tubes. Twenty microlitres of the blood was mixed with 380 μl eosin–acetone solution and incubated at room temperature for 5–10 min. Eosinophils were counted using a haemocytometer under a light microscope.

### 2.3 Soluble receptor assays

Commercially available ELISA kits were used to measure serum levels of sTNFR-I, sTNFR-II and sICAM-1 (R&D Systems, Inc., Minneapolis). ELISAs were carried out according to the manufacturer’s instructions and all serum samples were measured in duplicate. Sample dilutions were 1:25, 1:100 and 1:800 for TNFR-I, TNFR-II and sICAM-1, respectively. Concentrations for each soluble protein marker were determined from a serial-fold diluted standard. The minimum levels of detection for each assay were 6.25 pg/ml, 3.91 pg/ml and 7.81 pg/ml for TNFR-I, TNFR-II and sICAM-1, respectively.

### 2.4 Immunoglobulin assays

SEA and SWAP were prepared as described previously (Li et al., 1999); antigen-specific IgG4 and total IgG levels were measured by indirect ELISA. Briefly, ELISA plates (Nunc, Inc., Naperville) were coated with 5 μg/ml SEA or 10 μg/ml SWAP in 50 mM carbonate buffer (pH 9.6) and incubated overnight at 4 °C. Plates were washed three times with PBS (pH 7.6) containing 0.05% Tween 20^®^ and blocked using a PBS solution containing 1% BSA for 1 h at room temperature. After a further three washes, primary human serum samples were plated in duplicate in 1:100 and 1:200 dilutions for detection of IgG4 and total IgG, respectively, and again incubated at room temperature for 1 h. The plates were washed a further three times and incubated for 1 h at room temperature with Streptavidin–horseradish peroxidase (HRP)-labelled detection antibodies in dilutions of 1:400 for IgG4 (Zymed, Inc., San Francisco) and 1:100,000 for total IgG (Bethyl, Inc. Montgomery), respectively. After a final three washes, the detection reaction was developed using 3,3,5,5-tetramethylbenzidine (TMB) peroxidase substrate solution. After 30 min, the developed colour was stopped with 2 M H_2_SO_4_ and the OD was measured at 450 nm using a Bio-Rad^®^ ELISA reader. A blank PBS control was used on each plate and the OD values were adjusted for plate-to-plate variation by means of a positive internal standard.

### 2.5 Statistical analysis

All data were analysed using SPSS 13.0. Analysis-of-variance (ANOVA) was used to evaluate the overall significance between groups. Games–Howell post hoc tests were performed to assess the pair-wise differences in protein marker levels and immunoglobulin levels between the different clinically defined groups and multivariate nominal regression was used to estimate the strength of association for each soluble protein marker. Results are expressed as mean ng/ml for soluble protein markers and 95% confidence intervals (CIs) for each group. Antigen-specific IgG4 and total IgG results are expressed as ODs. A log transformation was applied to egg count data to normalise the distribution and was used as a measure of infection intensity (LnEPG). Correlations between markers and between LnEPG and eosinophil counts were analysed using the Pearson correlation coefficient. All figures display median protein marker or immunoglobulin levels for each clinical disease group.

### 2.6 Ethical considerations

Informed consent was obtained from all participants prior to blood collection. All patients who were stool-egg positive were offered a single dose of praziquantel at the recommended Word Health Organization (WHO) level (40 mg/kg). They were subsequently re-tested to ensure the infection was cleared. Ethical clearance for the study was obtained from the Medical Ethics Committee of Hunan Province and Queensland Institute of Medical Research.

## 3 Results

### 3.1 Soluble TNF receptors and ICAM-1 levels in the different clinically defined disease groups

**Fig. 1:**
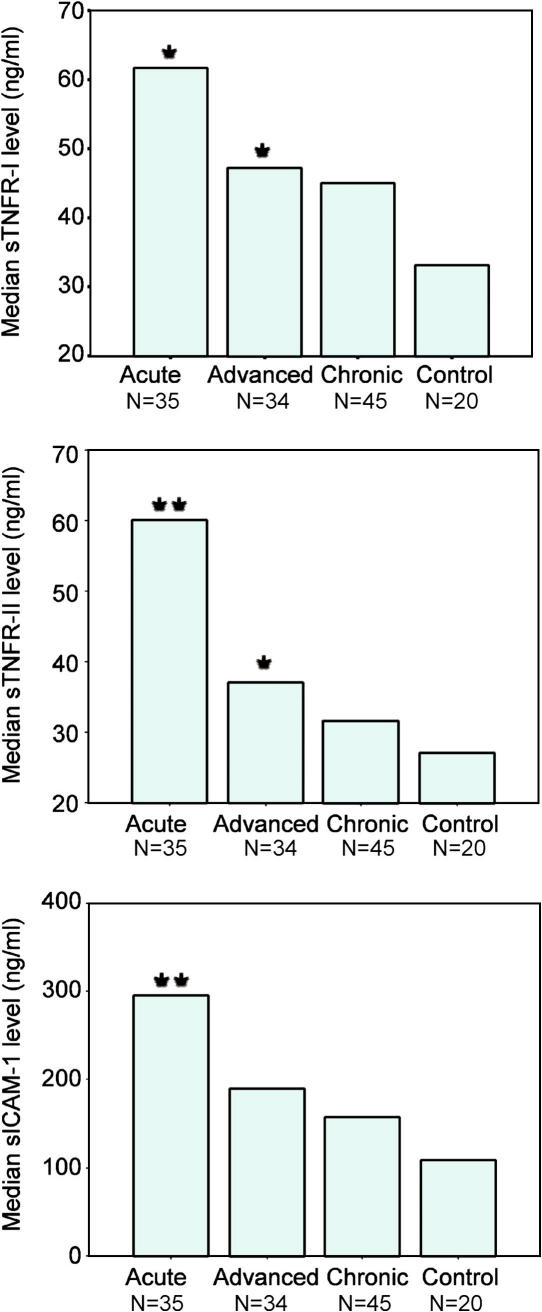
Median soluble fibrosis marker levels (ng/ml) in different *Schistosoma japonicum* clinical disease groups compared with non-exposed controls. Asterisks indicate significant mean differences between each disease group and controls as estimated using Games–Howell post-analysis of variance tests.

Elevated levels of sTNFR-I, sTNFR-II and sICAM-1 were observed in all clinically defined schistosomiasis groups compared with the control group (Fig. 1). Post hoc tests were used to identify significant differences between the means for each marker between each clinical disease group. The increase observed in chronically infected patients compared with the control group was not significant for any of the three protein markers (*P* = 0.0567; *P* = 0.0638 and *P* = 0.6996, respectively). Significantly higher mean levels of TNFR-I, sTNFR-II but not sICAM-1 were observed in advanced patients compared with controls (*P* = 0.0314; *P* = 0.00296; *P* = 0.1156, respectively). Acute patients demonstrated the highest levels of all three markers which were significantly higher than the control group (*P* = 0.0427; *P* < 0.000001; *P* = 0.00011, respectively) and significantly higher sTNFR-II levels were observed compared with advanced patients (*P* = 0.00008) and chronic patients (*P* < 0.000001). To identify markers most associated with disease, multivariate nominal regression was carried out. Likelihood ratio tests identified sTNFR-I to be a non-significant predictor of disease and it was subsequently omitted from the final model. The regression analysis showed sTNFR-II and sICAM-1 to both be significantly associated with acute infection in the presence of the other (*P* = 0.0001 and *P* = 0.0156, respectively). sTNFR-II showed the largest strength of association with disease (OR = 1.099; 95% CI = 1.047–1.154). Both markers were not significant for any other disease stage.

### 3.2 Soluble protein correlations, infection intensity and eosinophil counts

The Pearson correlation coefficient was used to estimate bi-variate correlations between the protein markers, infection intensity and eosinophil counts. All markers showed significant positive correlations with each other (Table 2), with sTNFR-I and sTNFR-II showing the highest correlation (Pearson correlation = 0.723; *P* < 0.00001) thus demonstrating a linear relationship between the two.

In the acute schistosomiasis group, highly significant positive correlations were found between eosinophil count and infection intensity (Pearson correlation = 0.477; *P* = 0.0072), and eosinophil count and the level of sICAM-1 (Pearson correlation = 0.363; *P* = 0.0014).

### 3.3 Immunoglobulin levels in the different clinically defined disease groups

**Fig. 2:**
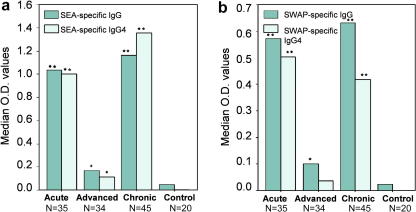
Antigen-specific IgG4 and total IgG levels in different *Schistosoma japonicum* clinical disease groups compared with non-exposed controls. Serum dilutions were 1:100 and 1:200 for the detection of IgG4 and total IgG levels, respectively. Asterisks indicate significant mean differences between each disease group and controls as estimated using Games–Howell post-analysis of variance tests.

ANOVA post hoc tests showed all SEA- and SWAP-specific total IgG and SEA-specific IgG4 levels were significantly higher in the acute, chronic and advanced groups compared with controls (Fig. 2). Antigen-specific immunoglobulin levels in acute and chronic patient groups were not significantly different from each other. SEA-specific total IgG measurements were significantly lower in advanced patients than in the acute and chronic disease groups (*P* < 0.00001; *P* < 0.00001, respectively) as were SWAP-specific total IgG levels (*P* = 0.0067; *P* = 0.0033, respectively). Though antigen-specific IgG4 levels were higher in acute and chronic patients than in the advanced patients (Fig. 2), there was no significant difference in SWAP-specific IgG4 between acute and advanced patients (*P* = 0.0643). The mean level of SWAP-specific IgG4 observed in advanced patients compared with controls was also not significant (*P* = 0.0891).

## 4 Discussion

Hepatic schistosomiasis is characterised by hepato-splenic inflammation due to an immunopathological reaction to deposition of schistosome eggs in the liver. The eggs cause an immune reaction mediated by eosinophils and macrophages and induce a delayed-type hypersensitivity response, resulting in granuloma formation. This reaction is a protective mechanism against the cytotoxic secretions from the eggs; however, long-term infections may result in portal hypertension and severe fibrosis of the liver. Although it is possible to detect some pathological features and mild forms of diffuse fibrosis using ultrasonography, it can be difficult, as in many cases there is no apparent sign of disease (Kardorff et al., 1996). Given the complexity of the liver damage and the variety of measurements, ultrasound readings require expert training in order to correctly determine disease severity and to monitor its progression. In addition, ultrasound can be impractical and expensive as a method of diagnosis and surveillance, particularly at a community level or if used on a large scale.

Previous studies have investigated biochemical markers in serum and plasma to evaluate their efficiency as potential markers of liver damage. Procollagen peptide levels have been shown to be elevated in schistosomiasis patients but drop significantly after praziquantel treatment rendering them ineffectual in monitoring disease progression (Kardorff et al., 1997). Further, overall sensitivity has been found to be low despite high specificity (Burchard et al., 1998). Hyaluronic acid (HA) has shown promising results correlating positively with ultrasound (US) gradings in schistosomiasis japonica (Li et al., 2000; Zheng et al., 2005) and schistosomiasis mansoni (Kopke-Aguiar et al., 2002). In addition, HA levels were not found to be associated with hepatitis infection, thus demonstrating its potential as a marker of fibrosis in patients with co-existing factors of liver inflammation (Eboumbou et al., 2005).

Previously, studies of intestinal schistosomiasis mansoni have implicated sICAM-1 in hepatic inflammation (Secor et al., 1994; Esterre et al., 1998; Mwatha et al., 1998) as it is known to play a role in granuloma formation (Ritter et al., 1993; Ritter and McKerrow, 1996) and in the modulation pathogenesis of hepatic and intestinal schistosomiasis (Jacobs and Van Marck, 1998). In this study, we found the highest levels of sICAM-1 to be amongst acute patients which supports previous findings that elevated expression of sICAM-1 is associated with egg deposition (Jacobs and Van Marck, 1998). Markedly elevated levels of sICAM-I were also observed in advanced schistosomiasis japonica patients diagnosed with periportal fibrosis (grade ⩾ II), a feature previously reported also in hepatic schistosomiasis mansoni (Mwatha et al., 1998). TNF-alpha is thought to aggravate hepatic schistosomiasis (Henri et al., 2002; Booth et al., 2004) and can induce shedding of TNF-alpha receptors TNFR-I and TNFR-II (Aderka et al., 1992; Aderka, 1996). The highly significant correlations of sTNFR-I with sTNFR-II in this study reflect this relationship. Further, high levels of sTNFRs were observed amongst the advanced schistosomiasis japonica patients studied here, implying they could play a key role in the development of hepatic fibrosis. It is noteworthy that the highest levels of these markers were observed in acute patients, thus implicating them in the pro-inflammatory response to granuloma formation and deposition in the liver. Levels of sICAM-1 were also found to significantly correlate with eosinophil counts in acute patients, supporting previous findings that sICAM-1 is crucial in the regulation of eosinophilic inflammation (Teixeira et al., 2001; Forbes et al., 2006).

SEA-specific levels of IgG4 and total IgG were significantly elevated in all disease groups compared with non-exposed controls. SEA- and SWAP-specific levels of IgG4 and total IgG were the same in chronic and acute schistosomiasis patients but were significantly higher than in the advanced group and thus are indicative of the presence of infection and an active immune response to the parasite. The levels were not significantly different between the acute and chronic patients and thus, though associated with the presence or absence of *S. japonicum* infection, they did not differentiate between the different clinical presentations of schistosomiasis japonica. However, given the high levels of these antibody isotypes in active infection, and the low levels of soluble protein markers observed in chronic patients, the results suggest a clear role for the latter in the granulomatous reaction to egg deposition in acute and advanced patients. The elevated levels of these markers in acute schistosomiasis demonstrate the severity of morbidity in these patients.

We believe this work presents the first investigation of sICAM-1 and sTNFR (I and II) levels in human schistosomiasis japonica and supports findings from previous studies of hepatic schistosomiasis mansoni (Esterre et al., 1998; Mwatha et al., 1998; Henri et al., 2002; Booth et al., 2004; Coutinho et al., 2005). In conclusion, there appears to be a strong correlation between sICAM-1, the sTNFRs and disease progression; the elevated levels of all three markers in acute and advanced schistosomiasis patients together reflect an intense inflammatory response. Determination of the levels in serum of sTNFR-II and sICAM-1 may serve as a useful tool for the detection of acute schistosomiasis, and the onset and progression of advanced disease.

## Figures and Tables

**Table 1:** Composition and definition of clinically defined schistosomiasis groups and controls

Disease phase	Number of individuals	Definition	Mean age (range)	Sex M/F	Period of water contact	EPG (range)
Acute	35	Documented recent water contact	19.4	34/1	10.4	225
		Tested positive for infection by Kato-Katz (Katz et al., 1972)	(6–65)		±SD 16.3 days	(6–2835)
		Tested positive for infection by serology (MPHC, 2001)				
		Clinical symptoms (fever, and/or other symptoms)				
		Eosinophilia (⩾15% of total leukocyte count)				
						
Chronic	45	Documented history of water contact	38.1	27/18	3.6	53
		Tested positive for infection by Kato-Katz	(7–68)		±SD 3.7 years	(4–516)
		History of re-infection				
		No or mild clinical symptoms				
						
Severe	34	Documented history of water contact	47.1	24/10	N/A	N/A
		Presence of hypersplenism and portal hypertension	(19–63)			
		Splenomegaly (Hackett degree ⩾ II)				
						
Non-exposed controls	20	From non-endemic region	39.2	14/6	0	0
		Tested negative for infection by Kato-Katz	(25–62)			
		Healthy during routine check-up				

N/A, not available; EPG, eggs per gram of faeces.

**Table 2:** Pearson correlation coefficients and two-tailed significances for correlations between markers, infection intensity and eosinophil counts

	sTNFR-II	sICAM-1	LnEPG	Eosinophil count
sTNFR-I				
Pearson correlation	0.723^b^	0.244^b^	0.164	−0.013
Sig. (two-tailed)	0.000	0.000	0.134	0.949
				
sTNFR-II				
Pearson correlation		0.368^b^	0.397^b^	−0.04
Sig. (two-tailed)		0.000	0.000	0.822
				
sICAM-1				
Pearson correlation			0.355^b^	0.363^a^
Sig. (two-tailed)			0.000	0.0442
				
LnEPG				
Pearson correlation				0.477^b^
Sig. (two-tailed)				0.0072

sTNFR-I, soluble tumour necrosis factor receptor I; sICAM-I, soluble intracellular adhesive molecule; LnEPG, log transformed data for eggs per gram of faeces.

a Significant at *P* < 0.05.

b Significant at *P* < 0.01.
